# Editorial: Updates in pediatric dentistry

**DOI:** 10.3389/fdmed.2025.1603591

**Published:** 2025-05-07

**Authors:** Jayakumar Jayaraman, Sreekanth Kumar Mallineni

**Affiliations:** ^1^ Private Practice, Richmond Pediatric Dentistry & Orthodontics, Richmond, VA, United States; ^2^ Pediatric Dentistry, Dr. Sulaiman Al Habib Medical Group, Ar Rayyan, Riyadh, Saudi Arabia

**Keywords:** dental atlas, supernumerary teeth, frenectomy, moderate sedation, root canal anatomy

**Editorial on the Research Topic**
Updates in pediatric dentistry

## Introduction

Pediatric dentistry is an ever-evolving field that continually integrates new diagnostic techniques, treatment modalities, and preventive strategies to ensure optimal oral health in children. The recent Research Topic of articles published in *Frontiers of Dental Medicine* under the theme “*Updates in pediatric dentistry*” showcases a broad spectrum of research, including advancements in diagnostic imaging, endodontic techniques, psychosocial influences on oral health, and clinical case management. This editorial summary synthesizes the key findings and contributions from these studies across six countries, highlighting their collective impact on pediatric dental care.

## Advancements in diagnosis and radiographic imaging

Supernumerary teeth, often associated with complications such as impaction, crowding, and delayed eruption, require precise radiographic localization for effective management. A narrative review on radiographic localization of supernumerary teeth emphasizes the superiority of three-dimensional imaging techniques, particularly cone-beam computed tomography (CBCT), over conventional two-dimensional radiographs. CBCT enhances diagnostic accuracy by providing a clearer spatial representation of supernumerary teeth, thus aiding in surgical planning and minimizing potential complications (Mallineni et al.). In a comprehensive dental atlas on the development and eruption of human teeth in the Chinese population, the author has provided crucial data on population-specific dental development patterns (Jayaraman). Such atlases serve as essential references for forensic dentistry, growth assessments, and orthodontic treatment planning, offering insights into variations in eruption sequences that may influence clinical decisions.

## Endodontic innovations and material sciences

Root canal treatment in primary molars is a challenging procedure, necessitating techniques that maximize efficiency while preserving tooth structure. An *in vitro* study comparing conventional manual files and rotary files in root canal preparation demonstrates that rotary instrumentation results in more consistent and conservative dentin removal (Vishwanathaiah). This finding supports the growing preference for rotary endodontics in pediatric dentistry, which can enhance treatment outcomes and reduce chair time. Another *in vitro* study evaluating the push-out bond strength of three root canal materials used in primary teeth investigates the adhesion properties of various endodontic filling materials (Özer et al.). The study concludes that some materials exhibit superior bonding to root canal walls, reinforcing their suitability for long-term endodontic success in children. Further research into tricalcium silicate-based materials and their bond strength to self-adhering glass ionomer cements highlights variations in bonding efficiency (BinSaleh et al.). These findings contribute to material selection strategies, ensuring durable restorations that withstand masticatory forces in pediatric patients.

## Malocclusion and dentofacial development

A study on the prevalence and contributing factors of malocclusion in children aged 7–8 years in Zhuang, southern China identifies genetic predisposition, oral habits including thumb sucking, tongue thrusting, and socioeconomic factors as significant influences on malocclusion development (Mai et al.). The study underscores the need for early orthodontic interventions to prevent complex treatment needs in later years. Similarly, an evaluation of anterior and overall tooth ratios in the Saudi population compared to Bolton's standards reveals discrepancies that may necessitate region-specific orthodontic assessments (Awawdeh et al.). These findings highlight the importance of considering ethnic and population-based differences when planning orthodontic treatments.

## Psychosocial and behavioral influences on pediatric oral health

Oral health outcomes in children are significantly influenced by psychosocial determinants, as demonstrated in a study on the role of parental education, socioeconomic status, and family dynamics in shaping children's oral hygiene practices and caries prevalence (Kopycka-Kedzierawski et al.). The research highlights the need for community-based interventions that address disparities in oral health knowledge and access to care. Another study examines the association between parental factors and children's behaviors during moderate sedation in pediatric dental care (Alanbari et al.). Findings indicate that parental anxiety, past dental experiences, and education levels directly impact a child's response to sedation. These results emphasize the importance of parental counseling and preparatory strategies to improve sedation outcomes. A study from northern Vietnam evaluates parental knowledge and practices in childhood caries prevention, revealing gaps in awareness and inconsistent adherence to preventive measures (Vu et al.). These findings call for intensified educational initiatives aimed at equipping parents with accurate information on dietary habits, fluoride use, and the importance of regular dental visits.

## Clinical case management and preventive strategies

**Figure 1:**
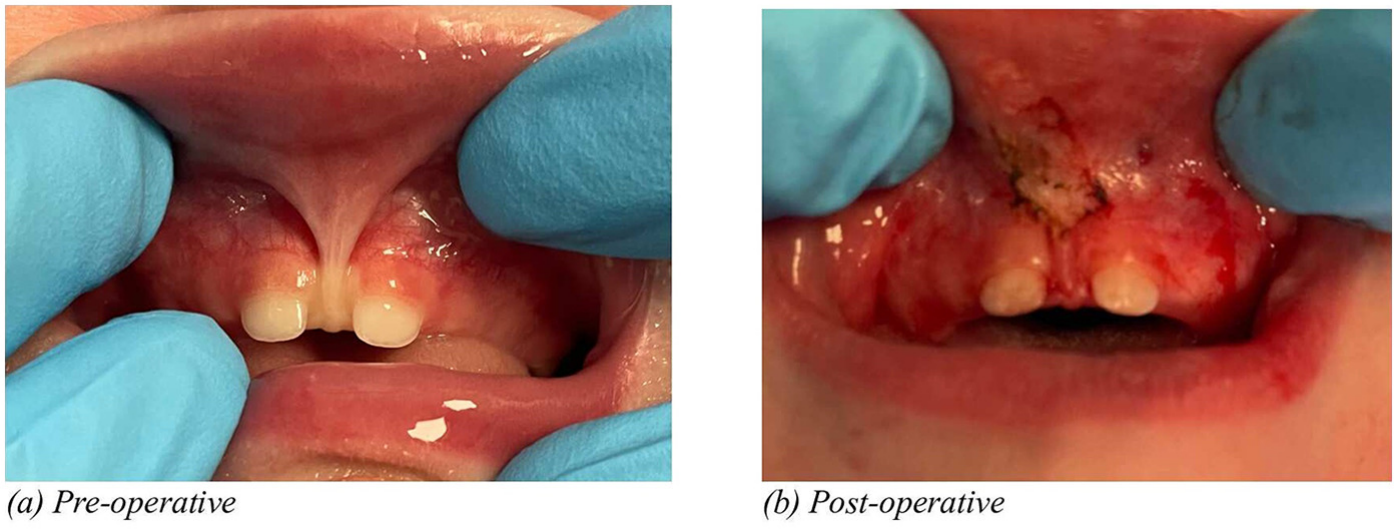
Pre-operative **(a)** and post-operative **(b)** illustrations of frenectomy in a six month infant performed by Laser.

**Figure 2:**
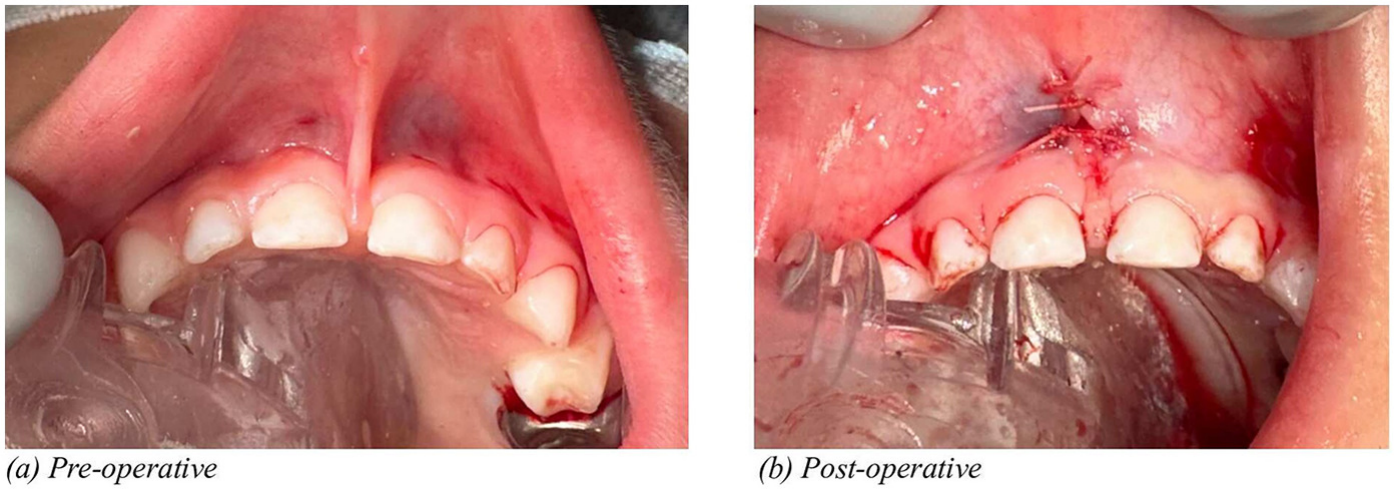
Pre-operative **(a)** and post-operative **(b)** illustrations of frenectomy in a three year old child performed using a conventional technique.

The case report on dental management of a child with congenital ichthyosis under general anesthesia highlights the complexities of treating pediatric patients with systemic conditions (Hino et al.). The report details modifications in treatment planning, emphasizing the necessity of interdisciplinary collaboration for safe and effective dental care. The “*Lift the lip*” screening guide, designed for dental professionals, aims to enhance early detection of oral health issues (Kotha). This initiative advocates for routine lip-lifting examinations to identify early signs of caries, and underlying systemic conditions, ultimately contributing to improved early intervention strategies. In addition, this study emphasizes the importance of evaluating soft tissue abnormalities, particularly frenum. Despite the growing acceptance of frenectomy, controversies remain regarding overdiagnosis and overtreatment. Several surgical techniques are available for frenectomy, with traditional and laser-assisted methods being the most commonly used. The laser technology has revolutionized frenectomy procedures and its advantages includes reduced bleeding due to hemostatic effects, minimal postoperative discomfort, no need for sutures in most cases resulting in faster healing with reduced scar formation (Figure 1). Alternatively, surgical incision may require suturing to optimize healing and minimize scarring (Figure 2). Some experts argue that a functional assessment should guide treatment decisions rather than anatomic classification alone (1). Standardized diagnostic criteria and long-term outcome studies are needed to refine clinical guidelines and future research should focus on providing evidence-based recommendations.

## Policy and public health implications

The article “*Allocating intricacies: pediatric oral health spotlight in the union health and well-being budget of India*” critically examines the allocation of financial and policy resources dedicated to pediatric oral health (Kumar et al.). The study highlights disparities in access to care and advocates for increased funding, improved infrastructure, and policy reforms to strengthen pediatric dental services in India.

## Conclusion

The collective findings from these studies underscore the importance of a multidisciplinary approach in pediatric dentistry. Advances in diagnostic imaging, endodontic techniques, and material sciences are enhancing clinical efficiency and treatment outcomes. Concurrently, research on psychosocial influences and public health initiatives emphasizes the role of education and policy in bridging gaps in pediatric oral healthcare. Moving forward, integrating these insights into clinical practice and policy frameworks will be pivotal in ensuring comprehensive and equitable oral healthcare for children worldwide.
